# Blood Flow and Continuous EEG Changes during Symptomatic Plateau Waves

**DOI:** 10.3390/brainsci8010014

**Published:** 2018-01-12

**Authors:** Natalie Kreitzer, Maggie Huynh, Brandon Foreman

**Affiliations:** 1 Department of Emergency Medicine, University of Cincinnati, Cincinnati, OH 45267, USA; 2 Department of Neurology and Rehabilitation Medicine, University of Cincinnati, Cincinnati, OH 45267, USA; huynhmt@ucmail.uc.edu (M.H.); foremabo@ucmail.uc.edu (B.F.)

**Keywords:** cerebral edema, meningioma, neuromonitoring

## Abstract

Benign meningiomas uncommonly lead to significant cerebral edema, with only a few cases previously reported in the medical literature. The present study describes the case of a 49-year-old female who had a meningioma resection. She subsequently developed malignant cerebral edema and had episodes that were initially concerning for seizure activity. However, transient blood flow changes concerning for intracranial pressure (ICP) crises, were demonstrated on electroencephalogram (EEG) as well as noninvasive cerebral blood flow monitoring. The present case highlights the importance of close monitoring in patients with post meningioma resection cerebral edema because of the possibility of ICP crises.

## 1. Introduction

Cerebral edema, defined as an increase in brain volume owing to an increase in brain water and sodium content is a life-threatening condition, and has a wide array of causes, including ischemic stroke, trauma, tumors, neurologic infections, high altitude, intracerebral hemorrhage, and metabolic disorders [1]. Although numerous treatments for both vasogenic and cytotoxic cerebral edema have been described in the literature, the mortality of patients with substantial cerebral edema secondary to malignancy is high. Meningioma, a common intracranial tumor, comprises 15–20% of all primary intracranial tumors [2]. Symptomatic meningioma resection has a 10% complication rate overall [3]. One such complication following meningioma resection is cerebral edema, which occurs in only 3.5% of cases [4]. 

We report a rare complication in a 49-year-old woman who developed malignant cerebral edema following resection of a meningioma in the atrium of the left lateral ventricle. The patient had paroxysmal clinical spells that appeared to be seizure-like in nature. Further, we report clarifying these paroxysmal clinical spells using noninvasive cerebral blood flow and electrocencephalography (EEG) monitoring to infer that spells may have been due to transient, critical compromises in cerebral perfusion, although direct intracranial pressure (ICP) measurements were never obtained. Plateau waves are characterized as sudden rise in ICP lasting several minutes and accompanied by paroxysmal neurological symptoms, e.g., depressed level of consciousness, pupillary dilatation, and dysautonomia [5,6]. When ICP increases, cerebral perfusion pressure (CPP) and subsequently cerebral blood flow (CBF) can decrease creating ischemia [7]. Non-invasively, CBF has been correlated with the cerebral flow index (CFI) of ultrasound-tagged near-infrared spectroscopy (UT-NIRS; ORNIM c-Flow^TM^). UT-NIRS has been shown to correlate with transcranial Doppler (TCD) changes during anesthesia induction and intubation, as well as acute Xenon single photon emission computed tomography (Xe-SPECT) changes during acetazolamide administration to healthy volunteers [8,9,10,11]. A more common neuromonitoring tool, EEG, may also be sensitive to changes in CBF, and has been used to detect ischemia during carotid endarterectomy, or after subarachnoid hemorrhage [12,13,14]. EEG and UT-NIRS have not been used together to characterize these events in a patient with cerebral edema following neurosurgery. 

## 2. Case Presentation

**Figure 1:**
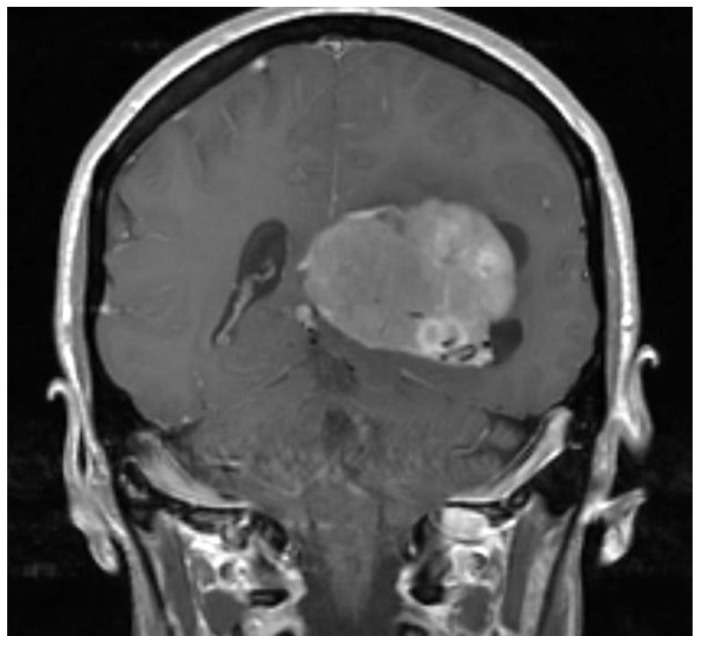
Pre-operative magnetic resonance imaging (MRI) demonstrates a large meningioma in the atrium of the left lateral ventricle.

**Figure 2:**
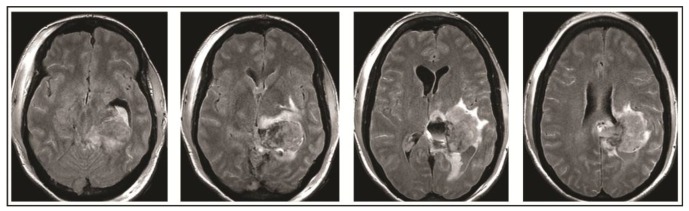
Post-operative day 1 MRI demonstrates 50% resection of left lateral ventricle meningioma.

**Figure 3:**
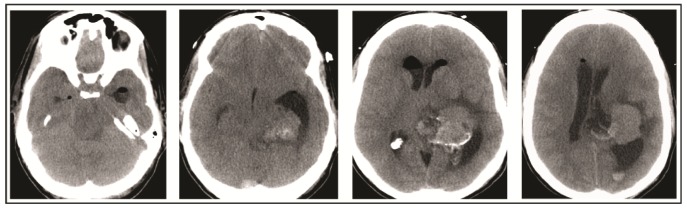
Head computed tomography (CT) performed on post-operative day 3 demonstrates cerebral edema and hydrocephalus.

**Figure 4:**
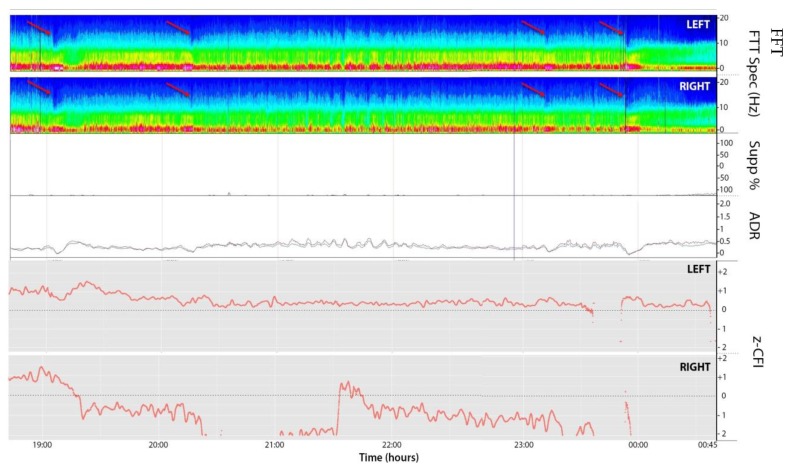
EEG compressed spectral array (CSA, 1–20 Hz; top two panels; left and right respectively), suppression ratio (Supp%), alpha-delta ratio (ADR; 8–14/1–4 Hz; 2 min moving average), and cerebral flow indices (CFI; bottom two panels, left and right, respectively) over an 8 h window. CFI are *z*-normalized to the mean for the entire time series, represented by dashed line. The red arrows denote periods in which the patient clinically experienced a depressed level of consciousness, pupillary dilatation, and dysautonomia. These periods correlate to attenuation of faster frequencies and small decreases in CFI. CSA: Compressed spectral array; CFI: Cerebral flow indices; Supp: Suppression; FFT: Fast Fourier Transform; ADR:Alpha delta ratio

A 49-year-old woman presented to her primary care physician after two months of headaches. A magnetic resonance imaging (MRI) demonstrated a large meningioma in the atrium of the left lateral ventricle, in addition to several other smaller meningiomas (Figure 1). She subsequently underwent approximately 50% debulking of the World Health Organization (WHO) grade 1 non-secretory transitional cell intraventricular meningioma [15]. The operation was a simple debulking surgery of standard duration, at six hours long. A halo self-retracting device was utilized for traction. Due to difficulty in visualization, it was aborted with only half of the tumor removed, with plans to later go back for additional resection. There was no significant intraoperative blood loss, and no significant intraoperative events. The surgical corridor used was the superior parietal lobule approach. An MRI on post-operative day (POD) 1 did not demonstrate significant edema (Figure 2). She initially had no neurologic deficits, but on POD 3, she developed transcortical motor aphasia. She also began having paroxysmal episodes of decorticate posturing, left pupillary dilation, diaphoresis, and gaze deviation. These episodes lasted several minutes, and occurred multiple times throughout the day. A non-contrast head CT (NCHCT) was performed, which demonstrated post-operative mass effect, cerebral edema, and an entrapped left lateral ventricle when compared to the MRI performed on POD 1 (Figure 3). Levetiracetam and dexamethasone were started, and hyperosmotic saline was given. Concern for seizures led to continuous EEG monitoring (cEEG), but did not demonstrate any ictal activity. UT-NIRS was used in conjunction with cEEG, and subsequent episodes were characterized by attenuation of faster frequencies and decrease in alpha-delta ratio on quantitative EEG, with relative decreases in CFI on UT-NIRS. These findings were suggestive of compromised cerebral perfusion pressure (Figure 4). This figure demonstrates in the left hemisphere, the CFI is maintained near its mean except during periods in which blood flow is lost completely toward the end of the recording period. In the right hemisphere, relative declines in blood flow are followed by a more profound loss of blood flow prior to decompression. Simultaneously, there is a decrease in faster frequencies on quantitative EEG in relation to changes in cerebral flow, noted by red arrows [16]. The paroxysmal episodes, denoted in Figure 4 as red arrows, were brief, initially occurred rarely, were first thought to be seizures, constituted an unusual presentation of cerebral edema, and the patient returned to clinical baseline in between with a high level of alertness. For these reasons, an external ventricular drain (EVD) and/or surgical decompression were not performed. 

**Figure 5:**
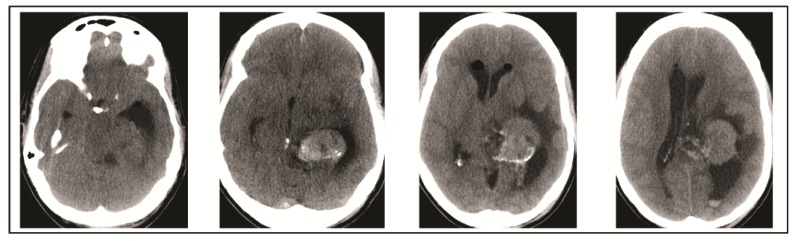
Head CT performed on post-operative day 7 demonstrates increase in cerebral edema, midline shift, and worsened hydrocephalus which prompted operative decompression.

**Figure 6:**
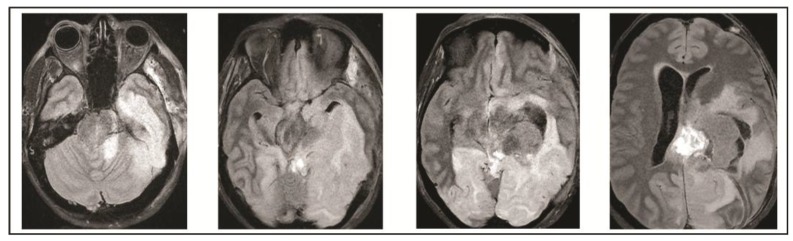
Final MRI on post-operative day 10 obtained demonstrated extensive infarcts secondary to vasogenic edema.

On the evening of POD 7, the patient had several spells clustered together (Figure 4). During the last episode, she had bilateral extremity shaking, progressive tachycardia, bilateral pupil dilation, and progression to coma without recovery. An urgent NCHCT demonstrated increased cerebral edema, midline shift, and trapping of the temporal horn of the left lateral ventricle, and the patient was taken to the operating room (OR) for decompression (Figure 5). Immediately prior to OR, cEEG showed burst suppression and decreased bi-hemispheric blood flow. Post-operatively, the patient’s neurologic exam demonstrated extensor motor responses and persistent coma; she had developed numerous herniation-related infarcts and persistent vasogenic edema (Figure 6) with eventual progression to brain death. 

This patient’s retrospective data was approved for research by the local institutional review board (IRB: Acute Neurological Injury Research Infrastructure: Observational Clinical Data Study; IRB Number 2015-0654). We did also attempt to contact the patient’s family members for consent to publish the case presentation, however, their contact information had changed in the interim since her death and they were unable to be reached.

## 3. Discussion

Malignant edema following a meningioma resection is a rare occurrence, but has been previously described in the literature. In one series of 376 patients requiring meningioma resection, 3.5% developed extensive edema requiring mechanical ventilation, reintubation, or decompressive craniectomy [4]. Ono et al. described two patients very similar to our case, in which edema developed following surgical resection. Both patients continued to have symptomatic edema for 3–4 weeks, and one of the patients required a second craniotomy during that time period [17]. Some authors have stated that meningiomas that interfere with venous drainage are at higher risk for significant cerebral edema following resection, and have recommended that a super-selective angiography be performed prior to resection [18]. In a retrospective study of 229 patients treated by single session or multisession radiosurgery or stereotactic radiotherapy four variables were associated with likelihood of edema development: tumor volume >4.5 mL, non-basal tumor location, tight brain/tumor interface, and atypical histology. However, in multivariate logistic regression, tumor volume and brain-tumor interface were found to be independent predictors of post-treatment edema [19]. 

In a patient with cerebral edema following meningioma resection, we were able to characterize transient, critical changes in non-invasively measured blood flow and slowing of the cEEG suggestive of compromised cerebral perfusion pressures. We hypothesize that these changes were secondary to elevated ICP, although direct measurement of ICP was never obtained in this case. Other case reports have described EEG changes noted prior to clinical changes in the setting of increased intracranial pressure, ranging from lateralized period discharges to bifrontally predominant generalized rhythmic delta, and finally suppression of EG activity [20,21]. However, these changes were described in scenarios by which progressive ischemia secondary to increased intracranial pressure had occurred, whereas our patient exhibited distinct episodic spells suggestive of transient ischemic events prior to herniation. Similar transient ischemic episodes were recently described in a perfusion-dependent patient following subdural hematoma [22]. The combination of EEG and UT-NIRS to characterize transient ischemic episodes related to likely elevated ICP has not been previously described. 

Patients with plateau-wave phenomenon have demonstrated impairment of CSF absorption and flow in previous literature [23]. Because this patient returned to full levels of alertness between spells, an external ventricular drain had not been placed, and ICP was not directly measured. In some cases, this may represent a beneficial management strategy for CSF diversion and direct ICP management. 

Malignant cerebral edema is rare following benign meningioma resection, but can lead to increased intracranial pressure, compromised cerebral perfusion, and ultimately to herniation. When evaluating a patient prior to surgery, those with a large tumor volume (>4.5 mL), non-basal tumor location, and a tight brain/tumor interface are likely at higher risk of post-resection edema. Post-operative findings of atypical histology represent a higher risk as well for post-resection edema. Paroxysmal neurological symptoms can be seen in the setting of seizures or decreased cerebral perfusion pressures and subsequently ischemia. Specifically, plateau waves are generally associated with decreased cerebral perfusion pressure, cerebral blood flow, brain tissue oxygenation, and cerebral oximetry [7,24]. Thus, it is plausible that changes induced during plateau waves could have also led to additional cytotoxic brain edema in addition to the vasogenic brain edema. 

## 4. Conclusions

We describe a patient in whom the combination of cEEG and UT-NIRS characterized transient episodes of decreased cerebral perfusion likely related to elevated intracranial pressure. In this patient’s case, cEEG provided additional information to global brain function when coupled with non-invasively measured CBF. In future, similar changes in UT-NIRS and cEEG associated with neurologic changes could initiate an earlier intervention. Further study, however, is needed to validate the use of non-invasive multimodal monitoring in patients at risk for cerebral edema and increased intracranial pressure. The value in such monitoring lies in the ability to detect compromise in cerebral blood flow and thus intervene medically and/or surgically in a timely fashion.
